# Benchmarking MeSH-augmented embeddings for biomedical document similarity

**DOI:** 10.1186/s13326-026-00365-6

**Published:** 2026-08-14

**Authors:** Rohitha Ravinder, Lukas Geist, Nelson Quiñones, Suhasini Venkatesh, Leyla Jael Castro, Dietrich Rebholz-Schuhmann

**Affiliations:** 1 ZB MED Information Centre for Life Sciences, Gleueler Str. 60, 50931 Cologne, Germany; 2 University of Cologne, Albertus-Magnus-Platz, 50923 Cologne, Germany

**Keywords:** Biomedical document similarity, Biomedical document retrieval, Document embeddings, Semantic similarity, Recommendation systems

## Abstract

**Background:**

The extensive volume of biomedical scientific literature requires efficient methods for retrieving relevant documents based on semantic technologies and biomedical concepts. While embedding-based methods have shown improvements over traditional keyword-based methods, the integration of domain-specific terminologies like Medical Subject Headings (MeSH) into these models remain underexplored.

**Methods:**

This study compares three hybrid methods that integrate MeSH-based annotations with document embeddings (called “pre-annotation”, “post-annotation” and“post-reduction”). We benchmark these hybrid methods against the following traditional methods: TF-IDF, standard neural embeddings (Word2Vec, fastText, Doc2Vec) and publicly available transformer-based models (BioBERT, SciBERT, SPECTER, SapBERT), using cosine similarity and Word Mover’s Distance (WMD) as evaluation metrics. The benchmark experiments are based on the RELISH corpus, a manually curated dataset of PubMed articles where experts have labeled pairs of documents with regards to their relevance to each other, providing a 2-class (relevant vs. non-relevant) as well as a 3-class (relevant, partially relevant, non-relevant) judgment.

**Results:**

Transformer-based models, particularly fine-tuned BioBERT and SciBERT align best with the expert judgements after fine-tuning. Among non-transformer methods, Doc2Vec and MeSH-based hybrid methods also perform well, demonstrating the benefits from combining structured biomedical vocabularies with embedding methods. Our experiments deliver extensive results showing that the baseline performance of 76–78% precision at position 5 can be achieved through almost all approaches, improvements of 2–4% with MeSH concepts can be achieved, but performances up to 90% is left to the fine-tuned large-scale public models.

**Conclusion:**

The performance gains from the integration of concepts may be underwhelming, however the benefits lie in the successful integration and benchmarking of structured vocabularies with embedding methods, the applicability of these techniques to aligning literature with other data sources via a controlled vocabulary and the potential for stronger performance on tasks and corpora where concept-based resources are better suited. All experiments have been conserved as a Dockerized pipeline, making the full benchmarking workflow reproducible and supporting future research in biomedical document retrieval.

**Supplementary information:**

The online version contains supplementary material available at 10.1186/s13326-026-00365-6.

## Introduction

Efficiently identifying relevant biomedical documents has been a long-standing challenge due to the high complexity and diversity of topics in this field. In particular, document relevance appears to be fundamentally tied to deep semantic interpretation in contrast to surface-level textual similarity and should benefit from capturing domain specific concepts and their associations. Medical Subject Headings (MeSH) [1], a community-based controlled vocabulary primarily in the medical domain, serves as an important means for standardizing and indexing the biomedical literature and thus for capturing conceptual relevance in documents of the biomedical domain. By contrast, in modern embedding-based approaches, the use of MeSH concepts as features indicating relevance remains underexplored.

The RElevance LIterature SearcH (RELISH) Corpus [2], a manually annotated dataset containing expert relevance judgments for biomedical document, provides an ideal collection for evaluating methods that expose document similarity. However, the documents in this corpus have not been annotated with explicit MeSH concepts, which can be interpreted as a challenge and an opportunity. Incorporating MeSH annotations through comparable methods should expose the implicit semantic relationships in RELISH and would provide new results for similarity assessments in this framework.

Traditional methods for assessing MeSH concept importance have relied on frequency-based metrics and co-occurrence patterns; however, these approaches fail to capture the nuances of semantic relationships between terms and their contextual surroundings in the document. Embedding-based approaches offer new alternative by transferring the document content in combination with MeSH concepts into the high-dimensional vector space, where semantic similarity can be easily evaluated. In benchmark experiments, the choice of the embedding architecture forms the key factor that fundamentally influences how MeSH contributes to the relevance of the documents according to the assessment.

Our research addresses these questions: How can MeSH concepts be effectively integrated into document embeddings to increase accuracy in the relevance assessments? Which parameters improved the effectiveness of selected embedding architectures when the document vector representation is augmented with MeSH concepts as features? How do the tokenization and representation methods as part of different embedding models influence the optimal approach for incorporating the standardized and structured vocabulary?

The remainder of the paper is organized as follows: Section “Background” provides background relevant to the scope of the study and reviews related work. Section “Methods” details our methodology, including hybrid integration strategies and the experimental setup. Section “Results” presents the results. Section “Discussion” discusses the findings and their implications and Section “Conclusion” concludes with future research directions.

## Background

Content-based recommendation systems play a crucial role in the field of information retrieval, particularly in domains such as biomedical research, where the sheer volume of scientific literature makes identifying relevant documents based on their inherent properties essential. Unlike collaborative filtering, which relies on user behavior and preferences, content-based systems analyze the intrinsic features of documents such as keywords, phrases, titles, author names and references to assess their similarity. By evaluating these features, content-based systems help researchers identify relevant studies, offering a more targeted and efficient search experience [3].

Traditional content-based recommendation systems in biomedical literature have largely relied on word-level similarity measures such as term frequency-inverse document frequency (TF-IDF) [4] which focus on keyword matching and basic term frequency analysis. While computationally efficient, they fall short in capturing the semantic context and deeper relationships between terms, which are crucial in the biomedical domain, where terminology often has specific nuanced meanings [5]. Early systems such as the CiteSeer [6] project laid the groundwork for academic literature retrieval by using TF-IDF and citation-based ranking to recommend relevant papers. Science Concierge [7] improved search relevance by integrating Latent Semantic Analysis (LSA) and the Rocchio algorithm, enhancing semantic understanding in search results. PURE [8] used Latent Dirichlet Allocation (LDA) to incorporate topic modeling, providing a more sophisticated approach to understanding document content beyond simple keyword overlap.Similarly, SciReader [9] personalized literature discovery by combining TF-IDF with LDA and PubMed-specific topic models, aiming to capture more complex semantic relationships within biomedical texts. Although effective at a basic level, these systems still rely heavily on surface-level keyword matching and statistical models, which fail to fully address the deeper semantic relationships and domain-specific contexts of biomedical terms. PubMed Related Articles (PMRA) service (currently known as “similar articles”), for example, uses probabilistic topic modeling based on term frequencies to suggest related articles, but still faces challenges in understanding the more intricate and specialized semantic connections that are inherent in the biomedical literature [10].

Advancements in Natural Language Processing (NLP) and neural network models, such as Word2Vec, Doc2Vec and fastText, have improved the ability to capture semantic relationships between documents by projecting words and documents into continuous vector spaces enabling document similarity assessments based on distributional semantics. For instance [11], showed improved performance in medical term similarity and classification tasks when domain-specific embeddings were trained on clinical notes and biomedical publications, while [12] highlighted the advantages of FastText-based models in article classification and sentence similarity by incorporating subword information. [13] evaluated word2vec models trained on PubMed and PubMed Central (PMC) to identify semantic relatedness of biomedical terms. It found that larger datasets identified more term pairs, but performance plateaued beyond 4 million vocabularies. Models trained on abstracts showed a higher correlation with reference standards, while those trained on article bodies identified more term pairs. Limitations include the use of specific reference standards, variations in word2vec implementations and the lack of cross-domain comparisons. However, these models rely on co-occurrence patterns in large corpora, which often fail to encode the hierarchical and ontological relationships inherent in structured biomedical vocabularies like MeSH. Consequently, embeddings may conflate terms with different contextual meanings or fail to capture synonymy and hierarchical dependencies crucial for precise document retrieval.

The use of embeddings has seen further innovation with transformer-based models such as BERT [14]. BERT’s contextualized embeddings capture word relationships within the broader context of an entire sentence, marking a significant leap over earlier word embedding techniques. In the biomedical domain, specialized models such as BioBERT [15] and SciBERT [16] have been trained on large-scale biomedical corpora, leading to significant improvements in tasks like document classification, information retrieval and semantic similarity analysis. These transformer-based models are particularly effective at understanding the intricacies of biomedical terminology, making them highly suitable for improving document similarity measures in this complex field. However, challenges remain in fully capturing the nuanced relationships within biomedical texts, especially in terms of understanding the interaction between content and domain-specific knowledge, which underscores the necessity for ongoing advancements in the field. A comprehensive benchmark on the RELISH corpus by [17] further confirmed that transformer-based models consistently outperform term-based approaches, yet none of the approaches integrated controlled vocabularies to augment the embeddings, a gap this study addresses.

Biomedical research is inherently complex, with specialized terminology that is often difficult to capture using general-purpose models. One of the primary tools for organizing and categorizing biomedical knowledge is the use of ontologies/controlled vocabulary. One such controlled vocabulary is MeSH. MeSH organizes biomedical knowledge through a hierarchical collection of MeSH concepts and are widely used by databases such as PubMed to categorize articles. These concepts capture both the explicit meaning of biomedical concepts and the subtle nuances of their relationships, making them a valuable resource for enhancing document similarity assessment in biomedical literature.

Several studies have explored the use of embeddings and ontologies for improving document similarity in biomedical literature. For instance [18] describes the MORE framework that integrates multiple ontologies with corpus-based word embeddings to enhance the semantic representation of biomedical concepts. By combining ontologies like MeSH and SNOMED-CT with word embeddings, MORE improves concept identification and clustering precision. However, its focus is primarily on refining concept representations without fully addressing the integration of content-based and semantic similarities at the document level. Similarly [19], generates literature-based biomedical concept embeddings on a large scale, evaluating their performance on tasks such as concept clustering and link prediction. While this approach benefits from large-scale corpora, it does not fully leverage semantic concepts like MeSH for semantic refinement, which limits its capacity to capture hierarchical relationships. On the other hand [20], enhances biomedical word embeddings by incorporating subword information with MeSH concepts, improving the precision of word representations, particularly for complex biomedical terminology. However, BioWordVec primarily focuses on word-level embeddings and does not integrate semantic and content facets at the document level.

### MeSH terminology

Several MeSH-related terms recur throughout this paper. To avoid the ambiguity noted in the literature between “words”, “terms”, “concepts” and “IDs”, we use them consistently as defined below.

#### MeSH term

A textual string appearing in a document either as a preferred label or one of its synonyms (e.g., “influenza”, “human flu”,“grippe”) that can be recognized and mapped to a MeSH concept.

#### MeSH concept

A controlled-vocabulary entry in MeSH represented by one or more MeSH terms (e.g., *Influenza, Human*). Each concept groups a preferred label and may be associated with one or more synonyms.

#### MeSH identifier (ID)

The unique alphanumeric code assigned to a MeSH concept (e.g., D007251 for *Influenza, Human*). The identifier serves as the canonical representation of the concept within our annotation and retrieval workflow.

#### Preferred label

The canonical name of a MeSH concept (e.g., *Influenza, Human*).

#### Synonym (lexical variant)

An alternative surface form that maps to the same MeSH concept and identifier as the preferred label.

#### MeSH top-level category

The highest level of the MeSH tree hierarchy under which concepts are grouped (e.g., *Diseases*, *Anatomy*, *Organisms*); referred to as a “category” throughout.

## Methods

### RELISH corpus

RELISH is an expert-curated dataset designed to facilitate research in document similarity within the biomedical domain. Developed by the RELISH consortium [2], it includes over 180,000 PubMed articles and contributions from more than 1,500 scientists. Each seed article is paired with 60 candidate articles according to their perceived similarity and are manually annotated for relevance as: *relevant*, *partially relevant* or *non-relevant* primarily by the original authors of the seed articles, ensuring highly reliable domain specific annotations. The corpus shows high inter-annotator agreement and no significant bias across annotator experience, research field or annotation effort [21].

We evaluated 18 embedding methods across three categories: standard text embedding methods, transformer-based methods and MeSH-augmented methods. Methods based on word and document embeddings were either trained on the RELISH corpus or applied in their pre-trained form. Transformer-based methods were finetuned and also used in their publicly available configurations. For the TF-IDF approach and the hybrid methods we used MeSH term identification to evaluate the influence of MeSH semantics on the embeddings (see below). All methods produce either word or document embeddings and were assessed based on their ability to capture semantic similarity between biomedical documents, using cosine similarity or Word Mover’s Distance (WMD) as similarity measures. The resulting scores were compared against expert relevance annotations from the RELISH corpus to measure how well each approach aligns with human judgment.

### Standard text embedding methods

#### Word2doc2vec

This approach uses the Word2Vec [22] framework to generate word embeddings, which are aggregated to create document-level embeddings. For each document, we compute the centroid (mean vector) of the embeddings for all words appearing in the title and abstract. We evaluate two variants: (1) a Word2Vec model custom-trained on the RELISH corpus and (2) the publicly available “word2vec-google-news-300” pre-trained model^1^. The model was implemented using the Gensim library [23] and both the skip-gram and Continuous Bag-of-Words (CBOW) training algorithms were considered.

#### WMD-Word2vec

This approach extends Word2Vec by using Word Mover’s Distance (WMD) to assess document similarity. WMD measures the minimum “distance” that words in one document must move to match words in another, with each word represented by its Word2Vec embedding [24]. The WMD score is inverted to provide a measure of similarity, where a higher score indicates greater similarity. As in the word2doc2vec method, we used both a custom-trained Word2Vec model and the pre-trained “word2vec-google-news-300” model for embedding generation. The Gensim library and its model.wv.wmdistance() function were used to compute WMD. Both skip-gram and CBOW architectures were evaluated, consistent with the word2doc2vec method.

#### Doc2vec

This method employs the Doc2Vec framework [25], an extension of Word2Vec that generates fixed-length vector representations for entire documents. Unlike Word2Vec, Doc2Vec learns an additional vector for each document to capture its semantic context. Both the dm (distributed memory) and dbow (distributed bag-of-words) variants were implemented using the Gensim library.

#### fastText

This method utilized the FastText framework [26], an extension of Word2vec that represents words as bags of character n-grams, enabling better handling of morphological variations and out-of-vocabulary words. Word embeddings for each word in the title and abstract in the RELISH corpus were generated using the FastText module from the Gensim library. Document-level embeddings were derived using the centroid aggregation method by averaging the word embeddings. For evaluation, we used both the pre-trained “fasttext/vectors-crawl/cc.en.300.bin.gz” model^2^ from Facebook’s FastText repository and a custom-trained FastText model tailored to the RELISH corpus with specific hyperparameters using both skip-gram and CBOW architectures.

### Transformer-based methods

We evaluated four transformer-based models adapted for scientific and biomedical domains: BioBERT, SciBERT, SPECTER and SapBERT. All models were accessed via the Sentence-Transformers library^3^ to generate sentence-level embeddings for titles and abstracts. BioBERT, pre-trained on PubMed and PMC texts, was tested in both its base (768-dimensional) and large (1024-dimensional) cased variants, specifically BioBERT-Base-cased-v1.1 and BioBERT-Large-cased-v1.1, developed by DMIS-Lab. SciBERT (scibert_scivocab_cased), trained on 1.14 M full-text Semantic Scholar papers, offers broader coverage of scientific terminology and produces 768-dimensional embeddings. SPECTER [27], designed for citation-based semantic similarity, generates document-level embeddings optimized for scientific literature and is included here due to its relevance to publication-level retrieval tasks. In addition, we evaluated SapBERT [28], a biomedical language model built upon transformer architecture and further pre-trained using a self-alignment objective over biomedical concepts and synonyms extracted from the Unified Medical Language System (UMLS). Unlike BioBERT and SciBERT, which rely primarily on masked language modelling objectives, SapBERT explicitly learns to place synonymous biomedical entities close together in the embedding space.

### MeSH-augmented methods

#### Whatizit-dictionary

This dictionary-based baseline method uses Named Entity Recognition to extract MeSH terms from biomedical texts, followed by TF-IDF weighting to quantify document similarity. MeSH term annotation is performed using Whatizit [29], a tool developed by the European Bioinformatics Institute (EBI) that combines rule-based pattern matching with machine learning techniques for biomedical NER. A minimal dockerized version of Whatizit was used in this study, which includes the MONQjfa component, a deterministic and nondeterministic finite automaton designed for Java. This version omits the web-based application and certain dictionaries available in the full version, making it lightweight and suitable for our study.

The annotated documents were represented using Term Frequency-Inverse Document Frequency (TF-IDF) to quantify the importance of MeSH terms within the corpus. Term Frequency (TF) captures how often a MeSH term appears in a given document, while Inverse Document Frequency (IDF) down-weights terms that appear frequently across many documents to emphasize more distinctive terms. The TF-IDF matrix was constructed across the full RELISH corpus using the extracted MeSH terms as features. Terms with zero document frequency across the corpus were excluded to reduce sparsity and noise. Pairwise document similarity was computed by applying cosine similarity to the resulting TF-IDF vectors. This method provides a lightweight, ontology-aware baseline to evaluate the effectiveness of purely term-centric methods relative to embedding-based methods in capturing semantic document relevance.

The term identifications were as well used in the following hybrid approaches to evaluate the influence of identified MeSH terms on the word and document embedding methods as presented above.

#### Hybrid methods

In this study, we explore three in-house hybrid strategies categorised as: pre-annotation, post-annotation and post-reduction. Each strategy combines previously described embedding techniques (Doc2vec, Word2doc2vec, WMD-Word2vec and fastText) with the aim of incorporating domain-specific semantic information from the MeSH controlled vocabulary. This integration is achieved either by annotating article titles and abstracts with MeSH terms before feeding embedding generation (pre-annotation), or enriching or replacing with MeSH-based semantic representations after the initial embedding process (post-annotation and post-reduction).

#### Pre-annotation

The pre-annotation hybrid method integrates dictionary-based NER with neural embedding models. Here, document titles and abstracts are first annotated with MeSH terms using an dictionary-based NER system, where recognized biomedical entities are tagged inline with their corresponding MeSH identifiers. This enriched text is then passed through the embedding models, allowing domain-specific concepts to be embedded alongside the original content. This strategy aims to inject biomedical terminology early in the pipeline; the full annotation process is described in Section “Data annotation”. We applied this method across four embedding approaches: *pre-annotation-word2doc2vec*, *pre-annotation-doc2vec*, *pre-annotation-wmd-word2vec* and *pre-annotation-fastText*.

#### Post-annotation

The post-annotation hybrid method enhances document embeddings by integrating semantic information after the initial embedding process. Models such as Word2Vec, WMD-Word2Vec and fastText are first trained on plain, preprocessed text to learn word-level representations. Following this, MeSH terms are identified in each document using previously annotated data and a MeSH concept index is constructed. This index maps each MeSH ID to the documents in which it appears, along with the corresponding lexical variants. For each MeSH concept, a representative vector is computed by aggregating the embeddings of the MeSH terms associated with that concept (e.g., synonyms and related expressions sharing the same MeSH ID). These MeSH concept vectors are added to the model’s vocabulary as semantic extensions. During testing, the MeSH identifiers associated with the identified MeSH terms are used to retrieve their corresponding concept vectors. These are combined with the original word-level embeddings via centroid aggregation to produce enriched document-level embeddings. This method is implemented across three embedding approaches: *post-annotation-word2doc2vec*, *post-annotation-wmd-word2vec* and *post-annotation-fastText*.

#### Post-reduction

This method follows a similar strategy to post-annotation but replaces MeSH terms in the text with their corresponding MeSH IDs rather than appending concept vectors. After generating word embeddings from raw text, a representative vector for each MeSH ID is computed by averaging the embeddings of its lexical variants. The original annotated text is modified by substituting MeSH terms with their MeSH IDs, enabling the model to directly embed standardized concept identifiers. This method is applied across three configurations: *postreduction-word2doc2vec*, *postreduction-wmd-word2vec* and *postreduction-fastText*.

### Evaluation metrics

To assess the performance of the embedding methods, we computed document similarity using cosine similarity between document-level embeddings, except for Word Mover’s Distance (WMD) based approaches, where cosine similarity was replaced by the WMD measure. Cosine similarity calculates the similarity of the document embeddings in the vector space representation, whereas Word Mover’s Distance measures a transitioning path for changing one document into the another one by replacing words and their word embeddings by similar words and embeddings.

Retrieval effectiveness was assessed using two standard information retrieval metrics: Precision@N and normalized Discounted Cumulative Gain (nDCG). Precision@N measures the proportion of relevant documents among the top N results, whereas nDCG accounts for the position of relevant documents in the ranking. Relevance judgments were evaluated under two labeling schemes:

- **2-class (binary relevance)**: *“relevant”* and *“partially relevant”* were merged into a single relevant category, compared against *“non-relevant”* category.
- **3-class (graded relevance)**: *“relevant”*, *“partially relevant”* and *“non-relevant”* were treated as distinct labels, allowing the evaluation to capture finer distinctions in biomedical document relevance.

### Experimental setup

**Fig. 1:**
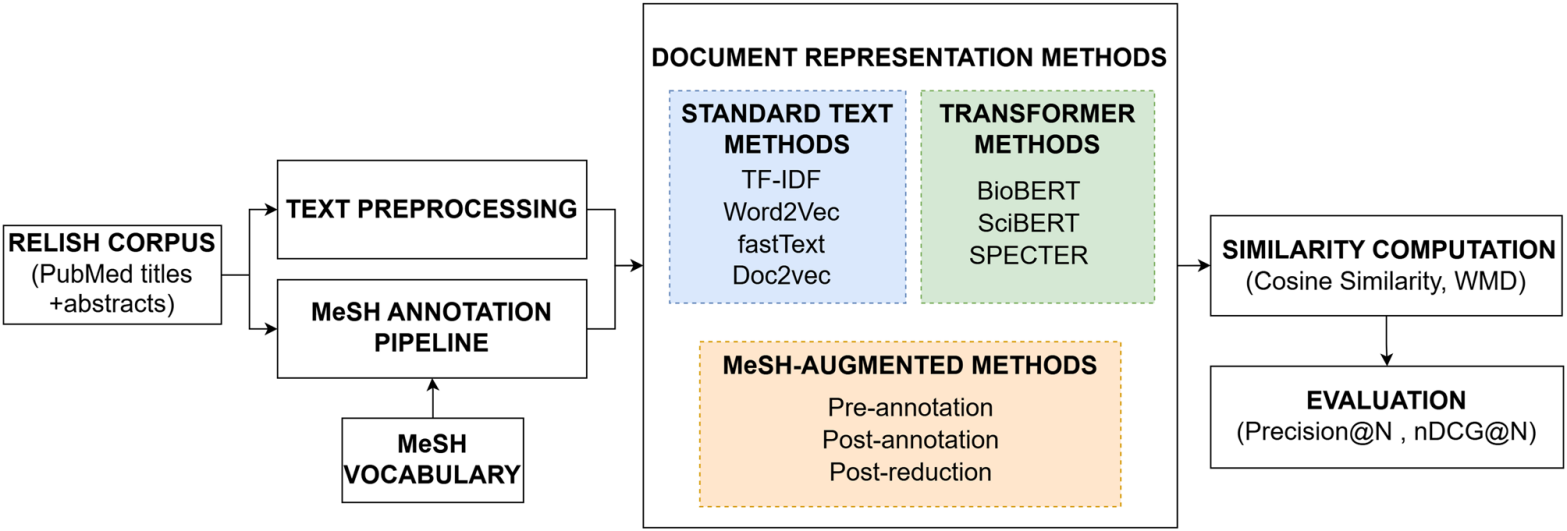
Overview of the experimental workflow. Documents from the RELISH corpus (PubMed titles and abstracts) are processed through two parallel pipelines: standard text preprocessing and MeSH annotation using the MeSH vocabulary. The resulting representations are fed into three categories of document representation methods: standard text methods (TF-IDF, Word2Vec, fastText, Doc2Vec), transformer methods (BioBERT, SciBERT, SPECTER) and MeSH-augmented methods (pre-annotation, post-annotation, post-reduction) and document similarity is computed using cosine similarity or Word Mover’s Distance (WMD). Performance is evaluated using Precision@N and nDCG@N

Figure 1 provides an overview of the experimental setup described in the following subsections.

#### Data retrieval

The RELISH dataset v1 was downloaded from its corresponding FigShare record [21] on January 24th, 2022. The dataset provided in a JSON format contains PubMed IDs (PMIDs) and document-to-document relevance assessments for each pair of documents. Using the BioC API, we retrieved an XML file containing the PMID, title and abstract of each document in the RELISH dataset. Any PMIDs that could not be retrieved or lacked titles and abstracts were recorded as missing. In total, we successfully retrieved 163,189 XML files. These XML files were then converted into a TSV format with three columns: PMID, title and abstract. The title and abstract text underwent further preprocessing explained in “Data preprocessing”. This TSV file was used as input for all approaches, except for the whatizit-dictionary method and the hybrid pre-annotation methods.

#### Data preprocessing

The preprocessing pipeline was tailored to each embedding-based approach, with steps defined based on established practices in biomedical text processing [11, 30–33]. For methods such as word2doc2vec, doc2vec, wmd-word2vec, fasttext, hybrid-post-annotation and hybrid-postreduction approaches, the titles and abstracts underwent several standard preprocessing steps. First, all text was converted to lowercase to standardize the input. Punctuation marks, except for hyphens were removed to preserve compound biomedical terms (e.g., “COVID-19”) and structural elements such as section headings (e.g., “BACKGROUND:”, “CONCLUSION:”, “METHODS:”), were eliminated, as they did not contribute to document similarity. Stop words were also removed to reduce noise. The text was then tokenized, splitting it into individual words or subwords. After preprocessing, the cleaned data was saved in .npy format for efficient handling during model training. For the BERT-based approaches, preprocessing was minimal. Only redundant whitespace and newline characters between the text were removed and the processed text was saved in a TSV format for input into the model.

#### Data annotation

The whatizit-dictionary and the pre-annotation hybrid methods rely on annotated text with the retrieved XML files serving as the input. To generate these annotations, document titles and abstracts were annotated with MeSH terms using minimal docker version of Whatizit, a dictionary-based biomedical entity recognition system. The annotation process began with the preparation of a controlled vocabulary dictionary from MeSH (2022 release). The MeSH RDF file was parsed to extract concept identifiers, preferred labels, synonyms and associated semantic types. Obsolete concepts were filtered out to ensure that only current, valid concepts are used for annotation. The extracted information was then converted into Minimal Word-Tagged (MWT) dictionaries, the dictionary format required by Whatizit. Each MWT entry links a MeSH identifier to its preferred label, synonyms and metadata such as semantic type.

**Fig. 2:**
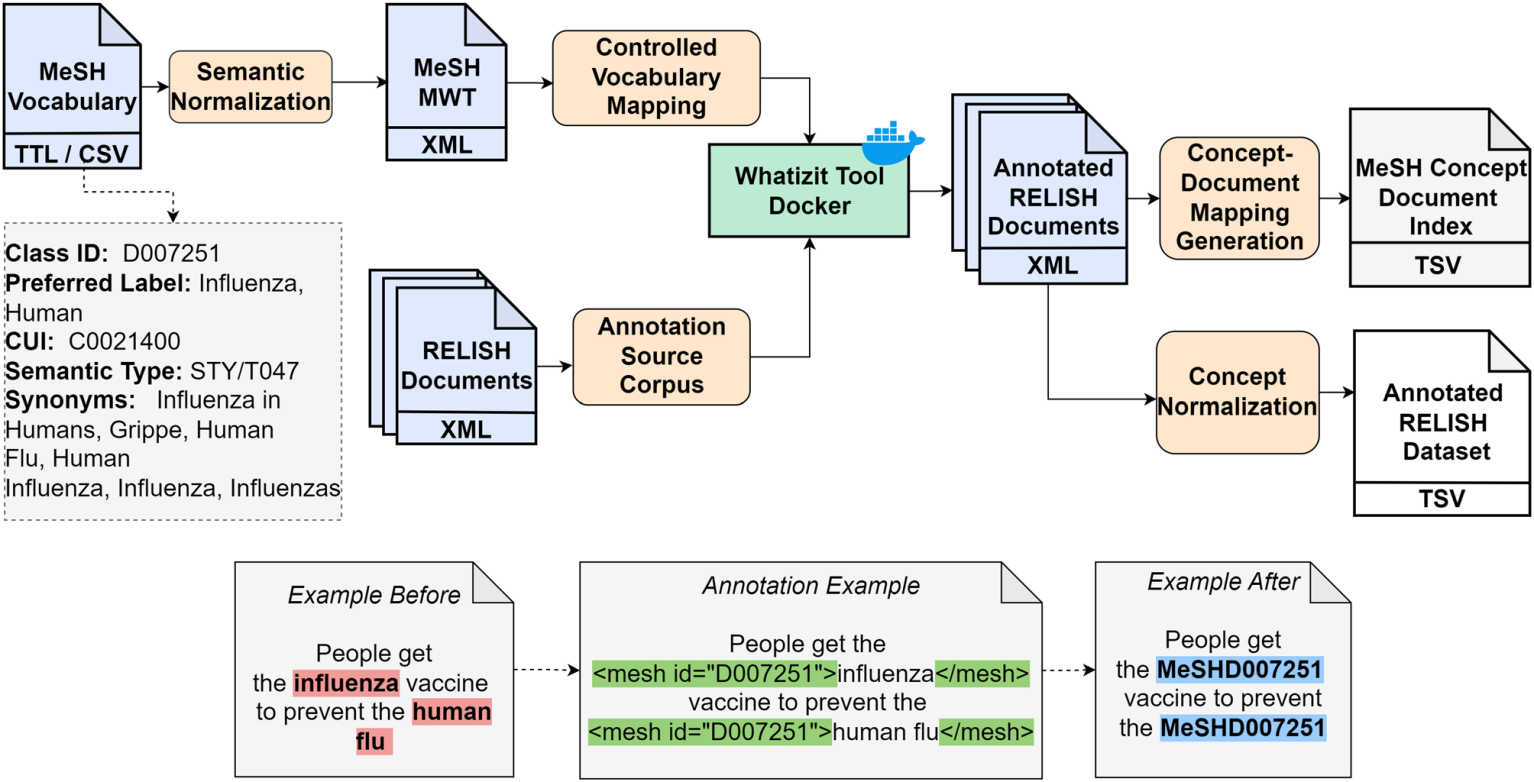
Overview of annotation workflow. The MeSH vocabulary (TTL/CSV) is semantically normalized into Minimal Word-Tagged (MWT) dictionaries, which are mapped to a controlled vocabulary and passed to the Whatizit Tool Docker alongside the RELISH documents (XML). Following entity normalization, annotated RELISH documents are produced and used to generate a MeSH concept-document mapping index (TSV). The example illustrates how recognized MeSH terms are normalized to the same MeSH concept and identifier (Influenza, Human; D007251) and how the corresponding MeSH identifier is subsequently used in the normalized corpus

During annotation, Whatizit scans document titles and abstracts and performs dictionary-based matching against entries in the MWT dictionary. Each entry contains a MeSH identifier together with its preferred label and synonym terms. Consequently, recognition is not restricted to exact matches of preferred MeSH labels; lexical variants explicitly listed as synonyms in MeSH are also identified and normalized to the same MeSH concept and identifier. For example, occurrences of “influenza”, “human flu” and “grippe” are all mapped to the MeSH concept Influenza, Human (D007251). The annotation process relies on dictionary entries and their associated synonyms rather than stemming, fuzzy matching, or context-dependent inference. Figure 2 illustrates the complete annotation workflow and provides an example of this normalization process.

The annotation output is generated in XML format, with recognized MeSH terms embedded directly within the document text. To preserve XML validity, special characters (such as &, ¡, ¿, ” and ’) are encoded prior to processing. The XML-annotated documents serve as the primary output of the annotation step and are used directly by the Whatizit-based retrieval approach. In addition, two derived resources are generated from these annotated XML files for use in the hybrid methods.

First, the annotated documents are normalized by replacing recognized concept mentions with their corresponding MeSH identifiers. The resulting normalized corpus is stored in TSV format, where each row represents a single document rather than a single annotation. Each row contains three fields: PMID, title and abstract. Within the title and abstract text, recognized biomedical entities are replaced by their normalized MeSH identifiers. This representation is used as input for the pre-annotation hybrid methods because these approaches require complete document text while also benefiting from concept normalization.

Second, a separate MeSH concept index is constructed from the normalized annotations. Unlike the TSV corpus, the concept index does not store document titles or abstracts. Instead, it organizes information by MeSH concept identifier and records the PMIDs of documents in which each MeSH concept appears together with the corresponding normalized MeSH identifier. This index is used for downstream concept-based retrieval and analysis.

#### Ground truth file generation

To evaluate the performance of document similarity models, we generated a ground truth dataset in TSV format. This dataset was derived from the original RELISH JSON file and contains three columns: PMID1 (reference article), PMID2 (assessed article) and relevance (relevance score between the two documents). For simplicity, document pairs were assigned a discrete relevance score: 0 indicating *“non-relevant”* articles, 1 indicating *“partially relevant”* articles and 2 indicating for *“relevant”* articles.

#### Data splitting

Prior to creating training, validation and test folds, we applied several preprocessing steps on the document pairs in the ground truth TSV file, which guided the split of the original RELISH corpus. Specifically, we filtered out pairs missing either a title or an abstract, removed duplicate assessed PMIDs and excluded reference articles that also appeared as assessed articles. After these filters, the dataset was split over 1,000 iterations. In each iteration, unique reference articles were split in a 9:1 ratio, with the larger portion allocated to training. The remaining 10% was further split evenly between validation and test sets, resulting in a final ratio of 90:5:5 for training, validation and test. These splits were applied to both the plain text and annotated text TSV files to maintain consistency across all data sources.

### Hybrid method pipelines

**Fig. 3:**
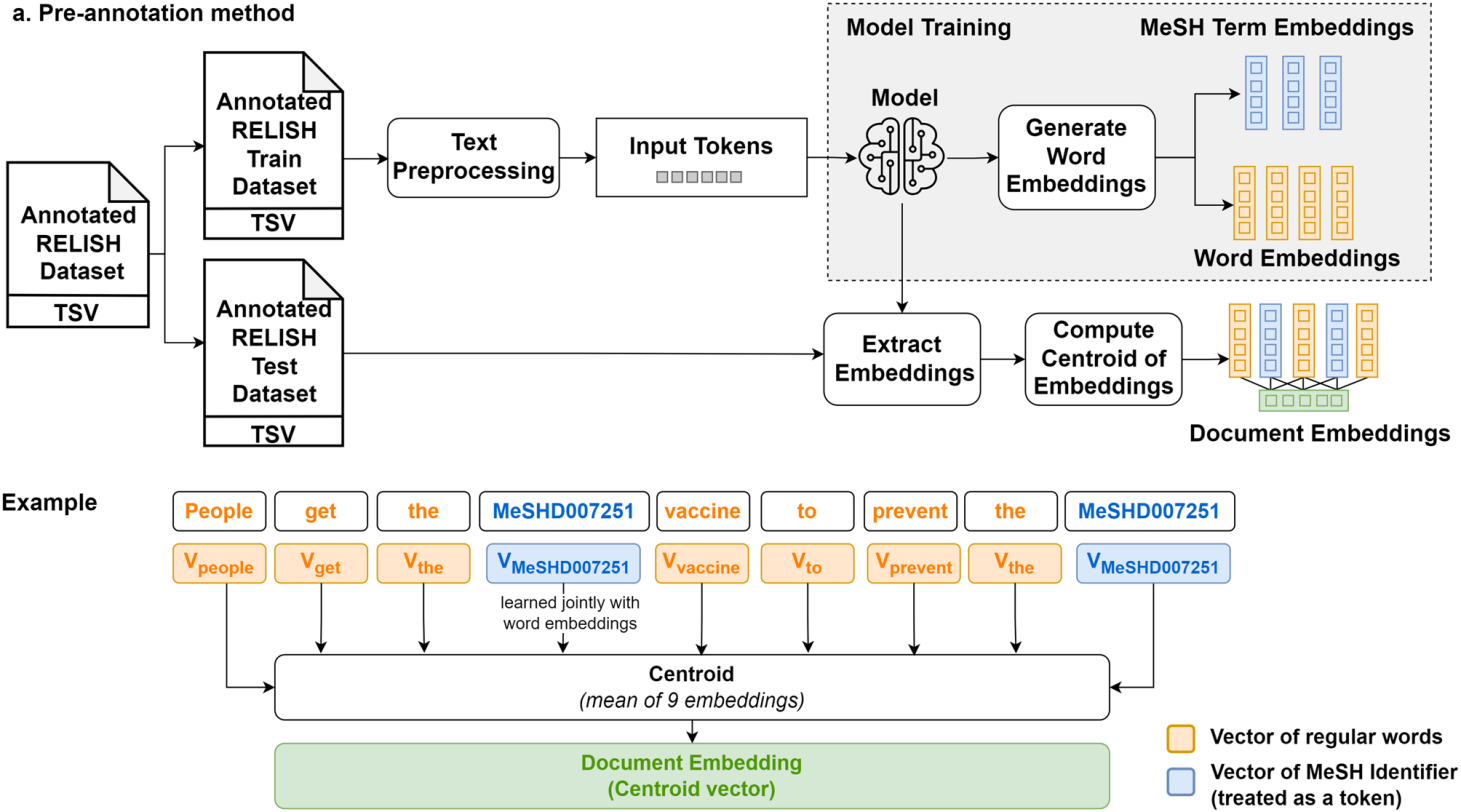
Pre-annotation hybrid pipeline. The annotated RELISH dataset (TSV) is split into training and test sets. During model training, preprocessed tokens from the annotated text are passed through an embedding model to generate word embeddings, including embeddings associated with normalized MeSH identifiers. Document-level embeddings are then computed by extracting and aggregating these embeddings via centroid calculation. The example illustrates this process at a whole word-level for clarity, the exact tokenization (e.g., whole-word vs. subword) depends on the specific embedding model used

**Fig. 4:**
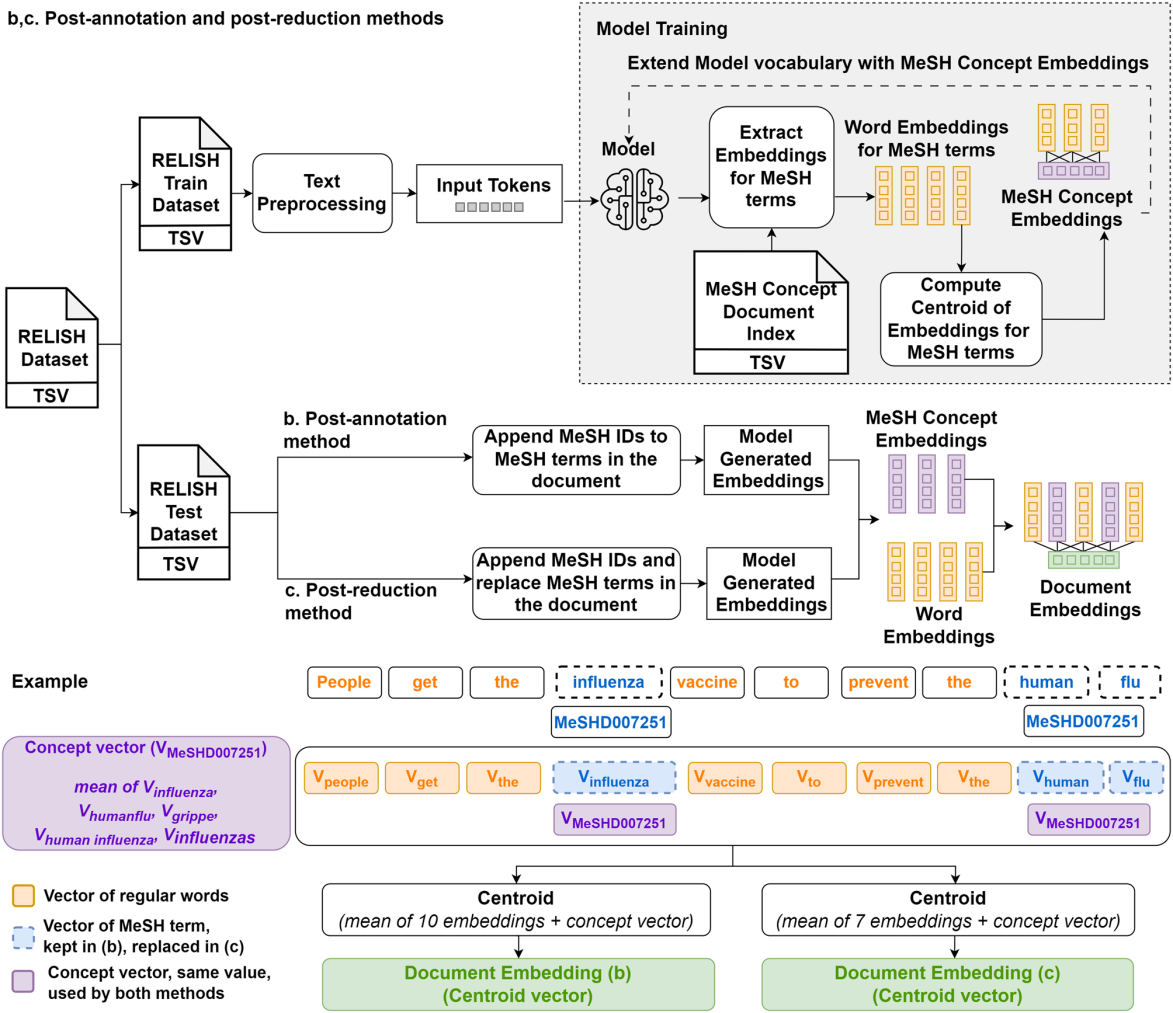
Post-annotation (**b**) and post-reduction (**c**) hybrid pipelines. In both strategies, the RELISH dataset is preprocessed and passed through an embedding model trained on plain text. MeSH concept embeddings are derived from the MeSH concept-document index and used to extend the model vocabulary. In post-annotation, MeSH IDs are appended to their corresponding terms in the document to enrich the final document embeddings. In post-reduction, MeSH terms are replaced by their corresponding MeSH IDs before embedding, allowing concept-level identifiers to be directly embedded into the document representation. The example illustrates this process at a whole word-level for clarity, the exact tokenization (e.g., whole-word vs. subword) depends on the specific embedding model used

Building on the annotation outputs described in Section “Data annotation”, Figs. 3 and 4 illustrate how MeSH annotations are integrated into the three hybrid embedding strategies.

#### Hyperparameter configuration and optimization

The evaluation workflow involved three stages: model training, hyperparameter optimization and performance evaluation. For all the approaches, except the Whatizit-dictionary and BERT-based models, hyperparameter optimization was performed using Optuna [34]. Each hyperparameter was assigned a predefined range of possible values, which Optuna explored to identify the best configuration.

Table 1 outlines the hyperparameter configurations used during model training. Word2Vec and fastText models were trained using either skip-gram (SG) or Continuous Bag of Words (CBOW) algorithms, while Doc2Vec models relied on either Distributed Memory (DM) or Distributed Bag of Words (DBOW).

**Table 1:** Hyperparameter configurations

Parameter	Range
sg / cbow	0 - 1
dm /dbow	0 - 1
vector_size	100 - 500 [Step: 50]
window	5 - 15
min_count	1 - 3
epochs	5 - 15
workers	1

Optimization was conducted over 100 iterations on the validation dataset, using Precision@5 as the objective metric. To ensure reproducibility, we applied a fixed seed of 42 for both model training and Optuna optimization, set the PYTHONHASHSEED environment variable to 42 and encapsulated the code in a Docker container. Once the optimal configuration was identified, the model was saved and evaluated on the test dataset. The final evaluation included computing cosine similarity for document pairs from the test ground truth file, along with Precision@N and nDCG@N for *N* values of 5, 10, 15 and 20.

#### Fine-tuning of BERT models

We fine-tuned BERT-based models using the Sentence-Transformers framework [35], which extends BERT to handle sentence or document-level similarity tasks. Sentence-Transformers implements a Siamese architecture, where two identical BERT encoders with shared weights simultaneously process document pairs to generate embeddings. Semantic similarity between documents is quantified by computing a similarity score between these embeddings. To optimize the fine-tuning process, we evaluated multiple loss functions: (i) Mean Negative Ranking (MNR) loss, which minimizes the distance between positive (similar) document pairs while pushing negative (dissimilar) pairs further apart, (ii) Contrastive loss, which ensures that the model learns to place similar documents closer together in the embedding space, while dissimilar documents are separated and (iii) Softmax loss, which treats document similarity as a multi-class classification task, assigning pairs to categories such as *“relevant”*, *“partially relevant”* or *“non-relevant”*. During fine-tuning, we monitored both training and validation loss using the validation dataset to track model performance and generalization. To improve generalization and reduce computation, we applied learning rate reduction on plateau, early stopping and dropout regularization. Models were trained for 3 epochs with a batch size of 16 and a dropout rate of 0.5.

## Results

### Corpus and annotation statistics

The RELISH Corpus consists of 163,189 unique articles, i.e., Medline abstracts from the biomedical domain in English. The document-to-document relevance assessments provided in the original RELISH dataset were transformed into the ground truth dataset described in Section “Ground truth file generation”. After applying the filtering steps outlined in Section “Data splitting”, 125,575 document pairs were retained. These pairs were further separated, yielding 113,017 pairs for training and 6,279 pairs each for testing and validation.

The MeSH (Medical Subject Headings) RDF Turtle (TTL) format for the 2022 release was sourced from the National Library of Medicine (NLM)^4^. The RDF TTL file contains the entire English MeSH vocabulary, detailing each MeSH concept’s unique identifier, preferred label, synonyms and hierarchical relationships to other terms. This vocabulary includes a total of 348,531 concepts, of which 34,525 MeSH concepts were present and annotated within the RELISH Corpus.

Table 2 summarizes the distribution of annotated MeSH concepts within the RELISH Corpus and, thus, the usefulness of MeSH concepts for similarity and relevance assessments. This information was used to set an appropriate range for the **min_count** parameter, ensuring that essential contextual information was retained. It is noteworthy that only approximately 10% of the total MeSH vocabulary was prevalent in the document set, indicating that a selective subset of MeSH concepts forms the core of semantic features in the annotated corpus.

**Table 2:** Overview over the occurrences of MeSH concepts across the RELISH Corpus

Frequency	MeSH concept count
* ≥ 5 *	20,564
* ≥ 4 *	22,224
* ≥ 3 *	24.413
* ≥ 2 *	28,120
* ≥ 1 *	34,525

**Fig. 5:**
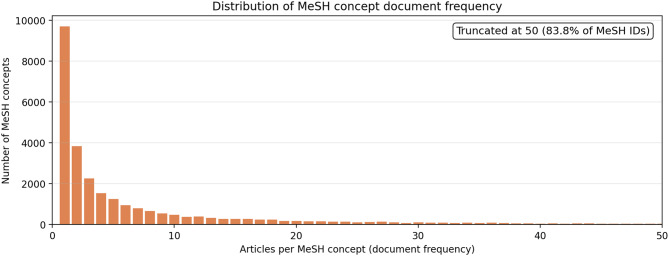
Distribution of MeSH concept document frequency in the RELISH corpus. For each annotated MeSH concept, the number of documents in which it occurs is counted. The distribution is strongly right-skewed: roughly 9,700 concepts occur in only a single article, whereas a small number of generic concepts appear in a large share of documents, with the most frequent concept occurring in 43,157 articles). The x-axis is truncated at 50 articles, which covers 83.8% of concepts. Only a small subset of the vocabulary is therefore broadly shared across the corpus, consistent with the long-tailed distribution of MeSH concepts typically observed in biomedical text

Figure 5 illustrates the distribution of MeSH concept frequencies across the corpus. The distribution is highly skewed: a relatively small number of concepts occur in a large proportion of documents, whereas most concepts appear only infrequently. This pattern is consistent with the long-tail distributions commonly observed in biomedical vocabularies and suggests that retrieval improvements derived from MeSH enrichment are likely concentrated around a subset of highly informative concepts.

### Effect of MeSH augmentation

**Fig. 6:**
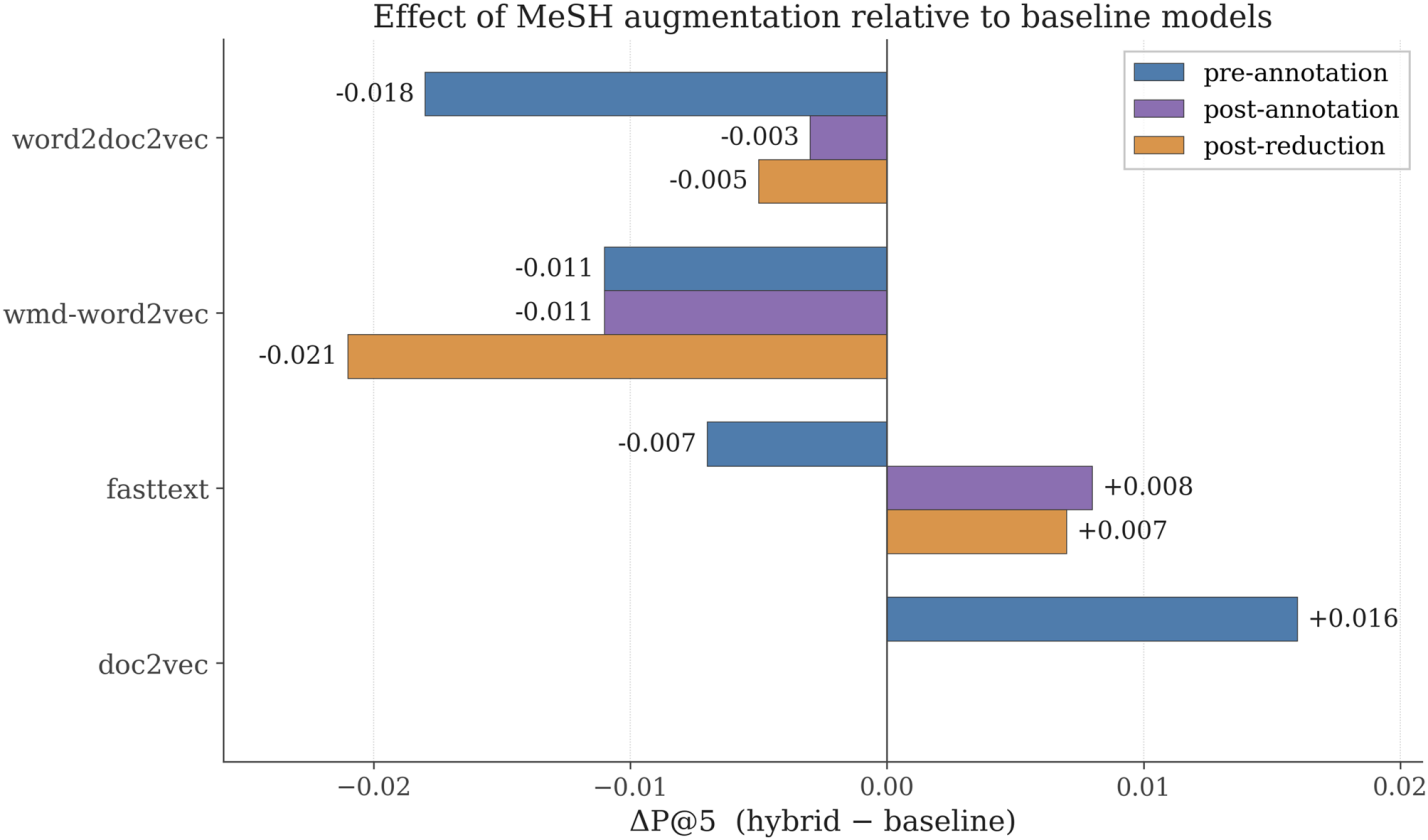
Effect of MeSH augmentation on retrieval performance measured by Precision@5 under the 2-class relevance setting. Values represent the difference in Precision@5 between each MeSH-augmented hybrid method and its corresponding baseline embedding model without MeSH enrichment. Bars are grouped by base architecture and colored by integration strategy; positive values (right) indicate improved retrieval, negative values (left) indicate a decrease

The primary research question of this study is whether enriching document representations with MeSH concepts improves the ability of embedding models to reproduce expert relevance judgments. Table 3 reports Precision@5 for each embedding architecture together with its MeSH-augmented variants under the 2-class relevance setting, while Fig. 6 summarizes the change in Precision@5 relative to the corresponding baseline model.

**Table 3:** Precision@5 under the 2-class relevance setting for each base embedding architecture and its MeSH-augmented variants. Bold indicates an improvement over the corresponding baseline; “–” denotes a strategy not applicable to that architecture

Base architecture	Baseline	Pre-annotation	Post-annotation	Post-reduction
Doc2Vec	0.823	**0.839**	–	–
fastText	0.800	0.793	**0.808**	**0.807**
Word2doc2vec	0.815	0.797	0.812	0.810
WMD-Word2vec	0.813	0.802	0.802	0.792

Across all architectures, the effect of MeSH enrichment is modest but architecture-dependent. The largest improvement is observed for Doc2Vec, where pre-annotation strategy increases Precision@5 from 0.823 to 0.839 (+0.016). FastText also benefits from MeSH enrichment with the post-annotation and post-reduction strategies improving Precision@5 by +0.008 and +0.007 respectivey. In contrast, Word2Doc2Vec and WMD-Word2Vec show little benefit and, in several configurations, experience small decreases in performance.

Importantly, the magnitude of these changes is small relative to the overall retrieval performance of the underlying architectures. Most methods remain within a narrow Precision@5 range of approximately 0.79–0.84, indicating that MeSH enrichment can improve biomedical document retrieval, but the gains are generally modest. The effectiveness depends more on the underlying representation than on the enrichment strategy itself, with document-level and subword-aware representations responding positively while word-centroid and transport-based representations show little benefit. We examine this interaction in the following section.

### Integration strategy and base architecture

At the level of strategy alone, the differences are small and follow a consistent ordering. Post-annotation is the safest strategy: it matches or exceeds the other variants for every word and subword-based method, as it retains the original word embeddings and adds concept vectors rather than replacing lexical information. Post-reduction, which substitutes recognized terms with their concept representations, is marginally lower in every case, indicating that the surface-level lexical signal still contributes once concept information is present. Pre-annotation is the most variable strategy: it is the most favorable option for Doc2Vec but the least favorable for Word2Doc2Vec, WMD-Word2Vec and fastText.

This contrast refects where each model forms its representation rather than by the timing of integration. Pre-annotation trains the embedding model directly on concept-normalized text, in which synonymous mentions are collapsed to a single MeSH identifier. For a document-level model such as Doc2Vec, this normalization enriches the document vector by making related articles share concept tokens and the method benefits. For word and subword-level models, the same operation is detrimental as it forces the model to learn representations for identifier tokens that lack the co-occurrence context (Word2Doc2Vec, WMD-Word2Vec) or the morphological structure (fastText) on which those representations depend. The post-hoc strategies avoid this problem because they derive concept vectors from embeddings that were already learned on natural text, which is why their effect is neutral-to-positive rather than disruptive.

### Influence of MeSH on document relevance

The modest improvements observed from MeSH augmentation suggest that only a subset of the controlled vocabulary contributes meaningfully to document relevance assessment. To better understand where this signal originates, we analyzed concept overlap within top-level MeSH categories and examined the lexical variation associated with annotated concepts.

**Fig. 7:**
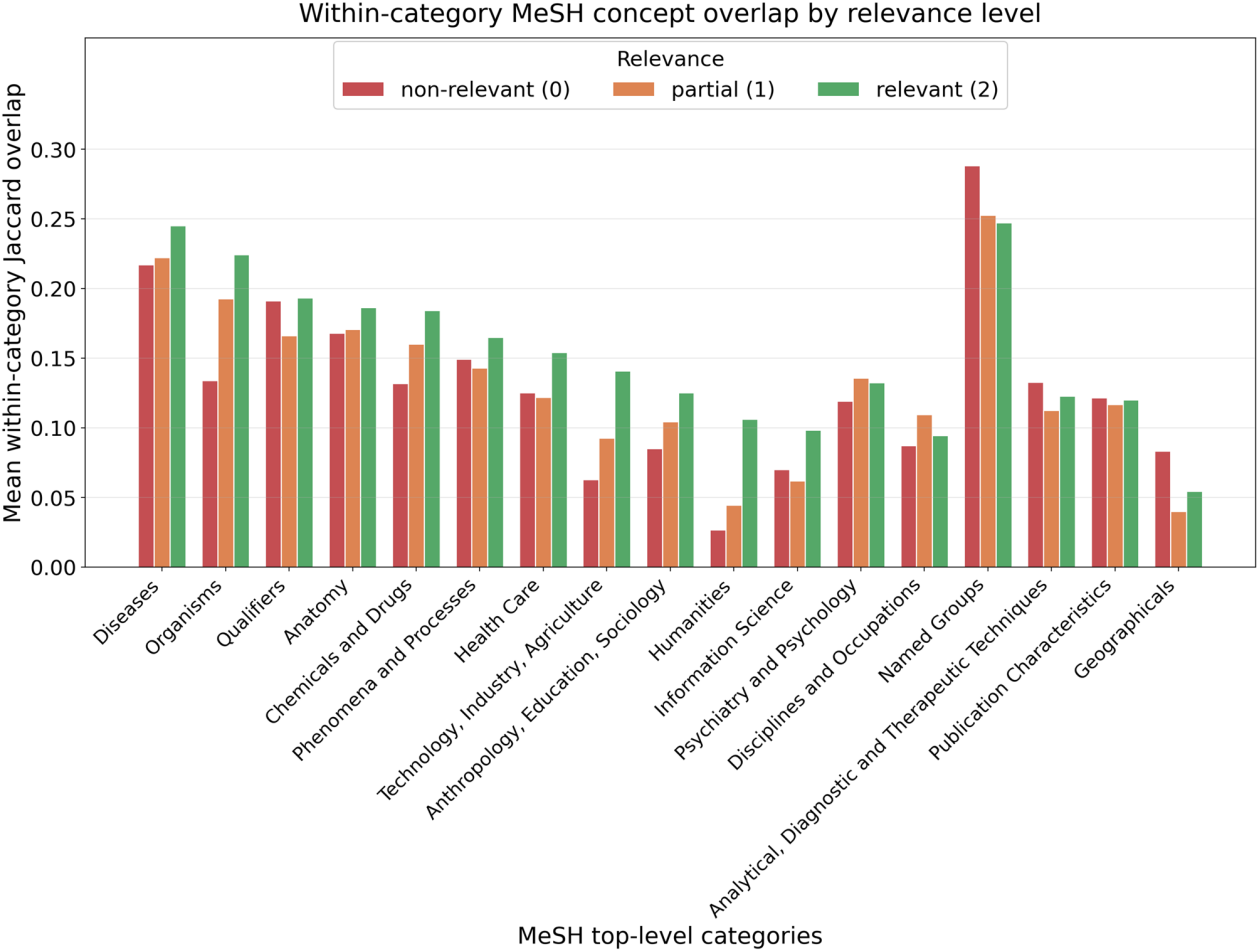
Mean within-category Jaccard overlap of MeSH concepts for document pairs in the RELISH test set, stratified by relevance level. Relevant document pairs generally exhibit greater concept overlap than partially relevant and non-relevant pairs, particularly within biomedical categories such as diseases, chemicals and drugs, organisms and health care, indicating that relevance information is concentrated within specific regions of the MeSH hierarchy

Figure 7 shows the mean within-category concept overlap for document pairs grouped by relevance level. Across most MeSH categories, relevant document pairs exhibit greater concept overlap than partially relevant or non-relevant pairs. The strongest separation is observed for categories such as *Organisms*, *Diseases*, *Chemicals and Drugs*, *Health Care* and *Humanities*, indicating that shared concepts within these domains are informative indicators of document relevance. In contrast, categories such as *Publication Characteristics*, *Geographicals* and *Named Groups* show limited or inconsistent discrimination between relevance levels. These findings suggest that the relevance signal captured by MeSH is concentrated within a subset of semantically rich biomedical categories rather than being distributed uniformly across the vocabulary.

**Fig. 8:**
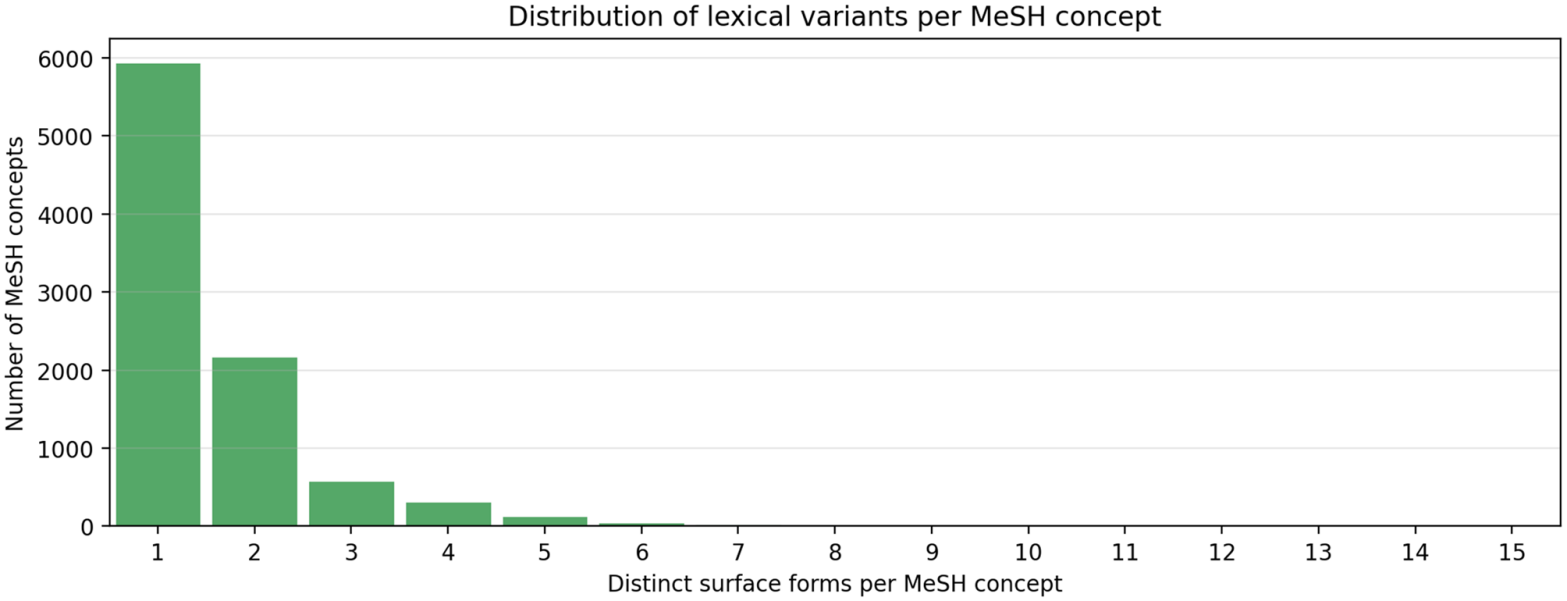
Distribution of lexical variant richness for annotated MeSH concepts in the RELISH test collection. The majority of concepts occur under a single observed surface form, while only a small fraction exhibit substantial lexical diversity. This limits the potential gains obtainable through synonym normalization alone

A complementary perspective is provided by the lexical variation analysis shown in Fig. 8. Although MeSH contains a large number of synonyms and alternative labels, lexical diversity within the RELISH corpus is limited. Approximately 64% of annotated MeSH concepts occur under only a single observed surface form, while the frequency of concepts associated with larger numbers of lexical variants decreases rapidly. Consequently, the opportunity for concept normalization to consolidate multiple synonymous expressions into a shared representation is restricted for a substantial proportion of the corpus.

### Comparative retrieval performance

Table 4 positions the MeSH-augmented approaches relative to the standard embedding models and transformer-based architectures, across the 2 class relevance setting using Precision@5 and nDCG@5. An additional file shows the complete precision@N and nDCG@*N* values for 2-class and 3-class distributions [See Additional file 1: Tables S1–S3].

**Table 4:** Comparison of retrieval performance under the 2-class relevance setting (*relevant* + *partially relevant* versus *non-relevant*).

Approach	Training type	P@5	nDCG@5
whatizit-dictionary	-	0.820	0.642
word2doc2vec	P	0.793	0.617
word2doc2vec	T	0.815	0.662
pre-annotation-word2doc2vec	T	0.797	0.648
post-annotation-word2doc2vec	T	0.812	0.657
postreduction-word2doc2vec	T	0.810	0.655
wmd-word2vec	P	0.777	0.636
wmd-word2vec	T	0.813	0.666
pre-annotation-wmd-word2vec	T	0.802	0.665
post-annotation-wmd-word2vec	T	0.802	0.666
postreduction-wmd-word2vec	T	0.792	0.657
fasttext	P	0.771	0.611
fasttext	T	0.800	0.656
pre-annotation-fasttext	T	0.793	0.653
post-annotation-fasttext	T	0.808	0.656
postreduction-fasttext	T	0.807	0.664
doc2vec	T	0.823	0.648
pre-annotation-doc2vec	T	0.839	0.660
BioBERT-base	P	0.782	0.623
BioBERT-base	F	**0.907**	**0.777**
BioBERT-large	P	0.802	0.628
BioBERT-large	F	0.890	0.754
SciBERT	P	0.793	0.624
SciBERT	F	0.875	0.730
SPECTER	P	0.864	0.732
SPECTER	F	0.879	0.766
SapBERT	P	**0.877**	**0.746**
SapBERT	F	0.887	0.775

Precision@5 (P@5) and nDCG@5 are reported for standard, MeSH-augmented hybrid and transformer-based approaches. Training type: P indicates models used with externally pre-trained weights, T indicates models trained on the RELISH corpus and F indicates transformer models further fine-tuned on the RELISH training split. Bold values denote the best overall performance, while underlined values indicate the best-performing configuration within each model family

For the 2-class distribution, *(relevant + partially relevant vs. non-relevant)* amongst the pre-trained BERT-based models, SapBERT outperformed other pre-trained baselines. However, unlike BioBERT, SciBERT and SPECTER, SapBERT benefited comparatively little from task-specific fine-tuning and did not achieve the same level of improvement observed for the other transformer models. Following fine-tuning, BioBERT-base achieved the highest overall Precision@N scores, followed by SciBERT and SPECTER. Among the non-BERT methods, document-level representations (*doc2vec* and the *hybrid pre-annotation-doc2vec* variant) are the most competitive but all standard and hybrid methods remain below the fine tuned transformers. The nDCG@N results show the same ordering: BioBERT-base achieved the highest overall ranking quality, although its advantage over SapBERT was relatively small, indicating that both models produced highly similar relevance rankings. Among the non-transformer methods, the wmd-word2vec family, including the post-annotation-wmd-word2vec variant, together with word2doc2vec, achieved the strongest ranking performance.

**Fig. 9:**
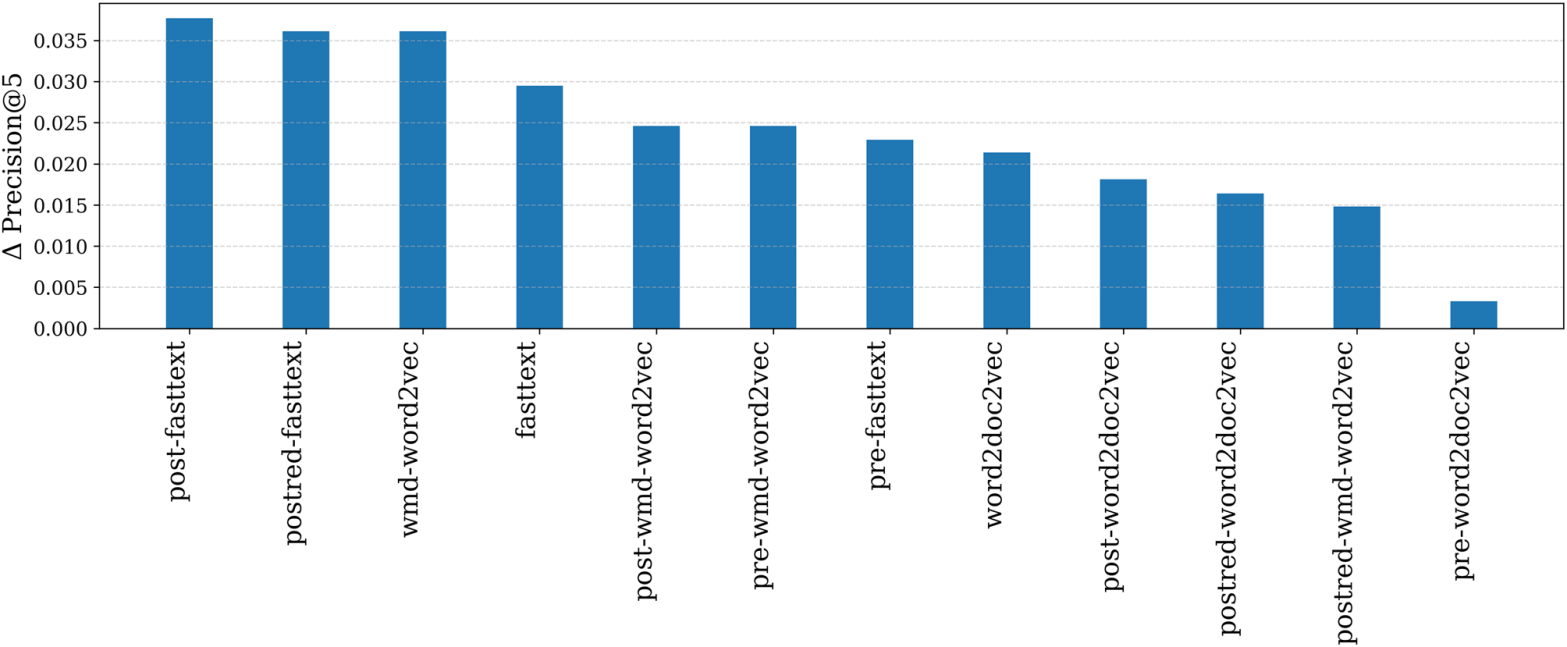
Absolute improvement in Precision@5 (*Δ*P@5) between trained and pre-trained hybrid embedding models for the 2-class relevance setting. Bars are sorted in descending order of improvement. FastText-based hybrid methods (post-fasttext, postred-fasttext) show the largest gains, while pre-word2doc2vec shows the smallest improvement, suggesting that annotation timing has a limited and architecture-dependent impact on retrieval precision

**Fig. 10:**
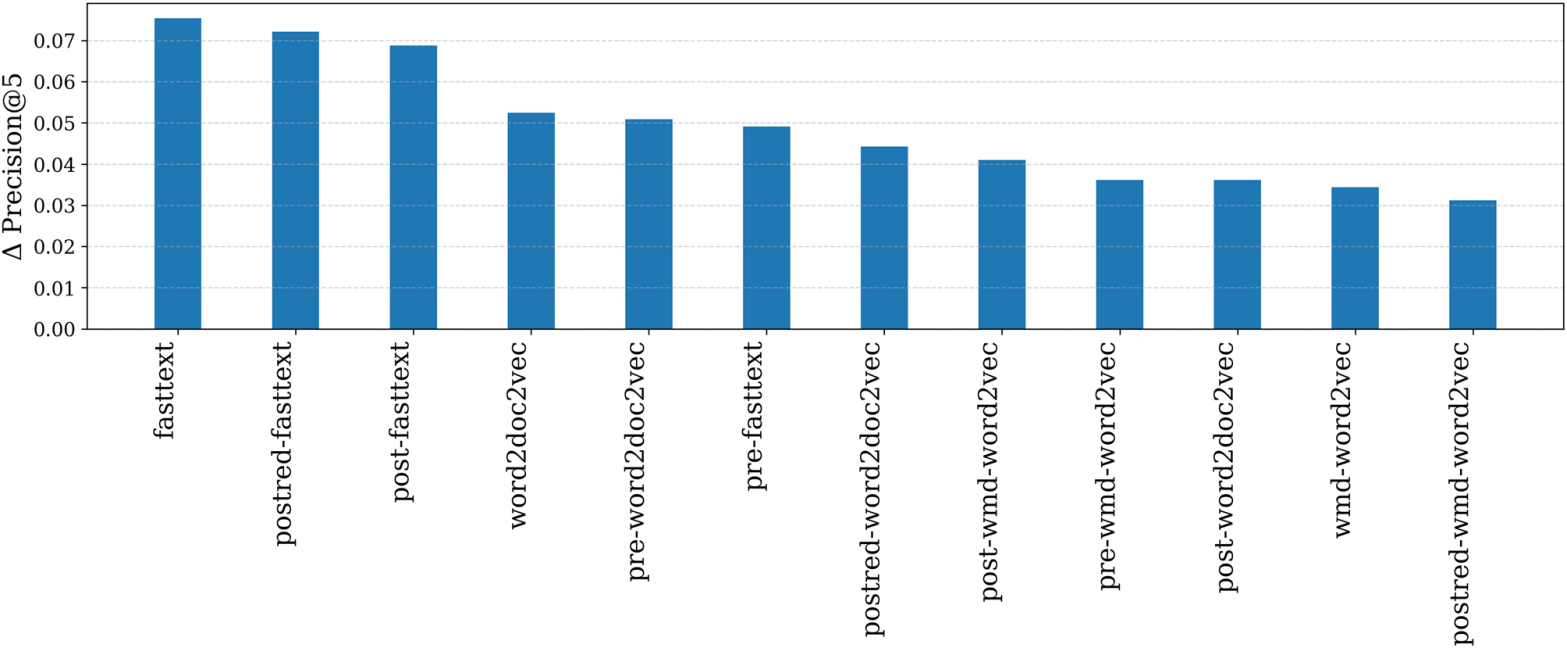
Absolute improvement in Precision@5 (*Δ*P@5) between trained and pre-trained hybrid embedding models for the 3-class relevance setting.Overall gains are larger compared to the 2-class setting, reflecting more improvement when distinguishing between relevant, partially relevant and non-relevant documents. FastText-based methods again lead, while wmd-based methods show the most consistent performance across annotation strategies

Figures 9 and 10 illustrate the absolute improvement in Precision@5 obtained by training hybrid embedding models compared to their pre-trained counterparts under the 2-class and 3-class relevance distribution settings, respectively. The observed gains from training these models are minimal and vary more across model architectures than annotation strategies. The small differences observed between pre-anotation, post-annotation and post-reduction approaches suggests that annotation timing has a limited impact on retrieval precision.

### Factors shaping retrieval performance

#### Relevance granularity

Expanding the task from a 2-class distribution to a 3-class distribution *(relevant vs. partially relevant vs. non-relevant)*, resulted in a noticeable drop in performance across all models. Precision scores were consistently lower, reflecting the added complexity of distinguishing *“partially relevant”* documents from both relevant and non-relevant articles. The overall trends were largely the same as those observed for the 2-class distribution, BERT-based models continued to dominate, while among the non-BERT models, *word2doc2vec* performed the best.

A point to note is that the class distribution does not directly affect nDCG. Minor variations observed are only due to the hyperparameters optimized for P@5 rather than class imbalance. Results are consistent across class distributions and BERT-based models achieve identical scores. For this reason, nDCG@N for 3-class distribution is not reported.

#### Vocabulary and out-of-vocabulary analysis

Additional file 1: Table S4 presents the optimal hyperparameter configurations for each approach trained on the corpus along with their corresponding vocabulary sizes and the out-of-vocabulary (OOV) counts.

**Fig. 11:**
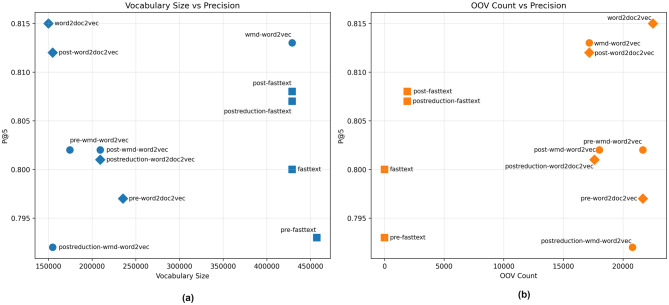
Vocabulary characteristics versus retrieval performance under the 2-class relevance setting. (**a**) Vocabulary size versus Precision@5 shows that larger vocabularies do not consistently lead to higher retrieval precision. wmd-word2vec achieves among the highest precision despite having one of the largest vocabularies, while word2doc2vec achieves the highest precision with a comparatively smaller vocabulary. (**b**) Out-of-vocabulary (OOV) count versus Precision@5 shows that higher OOV counts do not necessarily reduce performance, word2doc2vec and wmd-word2vec maintain the highest precision despite exhibiting the largest OOV counts, while fastText-based methods show near-zero OOV counts due to subword-based representation but do not outperform word-level methods. Marker shapes distinguish method types: circles indicate wmd-word2vec-based methods, diamonds indicate word2doc2vec-based methods and squares indicate fastText-based methods

Figure 11 relates Precision@N to both vocabulary size and OOV count across hybrid embedding configurations. Despite substantial variation in vocabularly size and OOV count, retrieval performance remains quite close. In particular, increasing vocabulary size does not lead to a large improvement in precision. Similarly models that eliminate OOV terms entirely such as *fastText* through n-grams and *doc2vec* through document level representations do not outperform word-level embedding approaches that exhibit higher OOV counts.

#### Similarity metrics

We compared cosine similarity and Word Mover’s Distance (WMD) using Word2Vec-based representations across *word2doc2vec* and *wmd-word2vec* models, including their hybrid variants. Although both approaches rely on the same underlying embeddings, they differ in how document similarity is computed, with *word2doc2vec* using centroid aggregation followed by cosine similarity and *wmd-word2vec* operating directly on word distributions via WMD. As seen from tables within Additional file 1, *word2doc2vec* outperforms *wmd-word2vec* even with a smaller vocabulary context. A similar trend is seen across post-annotation and post-reduction hybrid methods.

## Discussion

The primary objective of this study was to evaluate whether MeSH augmentation improves biomedical document similarity assessment, how MeSH concepts should be integrated into document representations and how the underlying embedding architecture influences the effectiveness of concept enrichment. Across all evaluated approaches, MeSH augmentation produced measurable but generally modest improvements in retrieval effectiveness. More importantly, the magnitude of these improvements depended strongly on the representation architecture, whereas differences between pre-annotation, post-annotation and post-reduction strategies were comparatively small.

The observed performance patterns are closely related to the characteristics of the RELISH Corpus. Within the RELISH Corpus, candidate documents are initially retrieved using PMRA, BM25 and TF-IDF, resulting in recommendation pools that are already highly relevant to the reference article. Consequently, the task is not one of identifying broadly related documents, but rather of distinguishing among documents that are already semantically similar. Under these conditions, even relatively simple embedding approaches perform competitively because they are required to capture subtle differences in relevance rather than large semantic gaps between documents.

The analyses presented provide further insight into why the gains from MeSH augmentation remain limited. Relevance signal was concentrated within a subset of MeSH categories, particularly those related to diseases, chemicals and drugs, organisms and health care, while other branches contributed comparatively little discriminatory information. At the same time, lexical variation among annotated concepts was relatively low, with most concepts appearing under only a single observed surface form. Together, these findings indicate that the amount of additional semantic information introduced through concept normalization is inherently constrained within the RELISH corpus.

The results also highlight the importance of representation learning in determining the usefulness of structured vocabulary enrichment. The largest differences in performance were observed across embedding architectures rather than across integration strategies. Doc2Vec and FastText benefited most from MeSH augmentation, whereas Word2Vec-based and WMD-based approaches exhibited little improvement regardless of how concepts were incorporated. This suggests that the ability of a model to exploit concept-level information is governed primarily by its representational capacity rather than by the specific mechanism used to inject MeSH concepts.

A further observation concerns the relationship between explicit and implicit concept representations. Hybrid approaches introduce biomedical concepts explicitly through MeSH term annotation and concept normalization, whereas transformer-based models acquire conceptual knowledge implicitly through large-scale pretraining on biomedical literature. The strong performance of BioBERT, SciBERT, SPECTER and SapBERT indicates that contextual language models already capture much of the conceptual structure represented by controlled vocabularies. Nevertheless, explicit concept augmentation remains valuable for lightweight and interpretable retrieval systems, where controlled vocabularies provide a transparent mechanism for incorporating domain knowledge without requiring large-scale pretraining.

This distinction is also relevant in the context of modern large language models (LLMs) and retrieval-augmented generation (RAG) systems. Contemporary foundation models are trained on corpora that are substantially larger than those used to develop domain-specific transformers such as BioBERT or SciBERT and may therefore capture a broader range of biomedical concepts implicitly through scale alone. However, the objective of the present study is not to evaluate generative retrieval systems, but rather to investigate the contribution of an explicit controlled vocabulary layer to document similarity assessment. In this setting, MeSH augmentation offers a transparent and interpretable mechanism for introducing domain knowledge into retrieval pipelines, whereas the conceptual knowledge encoded within large language models is largely implicit and difficult to isolate. The findings therefore complement, rather than compete with recent advances in LLM-based retrieval by examining the specific contribution of structured biomedical vocabulary information.

Finally, the present work exploits only part of the value offered by MeSH as a structured biomedical resource. The hybrid approaches primarily treat MeSH as a collection of concept identifiers and do not explicitly leverage its hierarchical organization. However, the uneven distribution of relevance signal across MeSH branches suggests that the structure of the vocabulary itself may contain additional information that remains unused.

Certain limitations should be considered when interpreting these findings. First, the evaluation was conducted exclusively on the RELISH benchmark, whose candidate generation process yields highly similar document pools and may reduce the observable impact of concept augmentation. Second, although hybrid approaches occasionally produced meaningful improvements, these gains were generally small relative to the additional complexity introduced by annotation and concept integration pipelines. Finally, retrieval effectiveness was influenced by the definition of relevance itself. Performance consistently decreased under the 3-class distribution, reflecting the ambiguity associated with partially relevant documents and demonstrating that evaluation outcomes depend not only on model quality but also on the granularity of relevance judgments.

## Conclusion

This study investigated whether MeSH-based concept enrichment improves biomedical document similarity assessment within the RELISH corpus and examined how different integration strategies interact with modern embedding architectures.

Across all experiments, embedding-based methods consistently outperformed traditional approaches such as TF-IDF and classical word embeddings. Transformer-based models, particularly BioBERT and SciBERT, achieved the strongest overall performance, confirming the value of contextualized representations for biomedical text. At the same time, several non-BERT approaches, including Doc2Vec and hybrid models that incorporate MeSH annotations, performed competitively, demonstrating that simpler representations remain effective under constrained retrieval settings.

Our analysis further shows that performance is strongly influenced by annotation design and evaluation choices. Moving from a binary to a 3-class relevance scheme led to a consistent drop in precision, reflecting the inherent ambiguity of partially relevant documents. This suggests that relevance granularity plays a critical role in model evaluation and that simpler relevance schemes may offer more stable and interpretable results. In addition, the consistent advantage of cosine similarity over Word Mover’s Distance indicates that similarity metrics are sensitive to preprocessing decisions and that transport-based measures may offer limited benefits in heavily preprocessed biomedical corpora.

Finally, this work emphasizes the importance of reproducibility in biomedical NLP research. By providing a fully Dockerized experimental setup, we enable transparent replication and fair benchmarking of models and evaluation strategies. We hope that this work supports more rigorous and reproducible comparisons in biomedical document retrieval and informs the development of practical, scalable recommendation systems that balance performance and interpretability.

## Electronic supplementary material

Below is the link to the electronic supplementary material.

Supplementary Material 1. Complete benchmark results and vocabulary analysis. Contains evaluation results referenced in the main text, including full Precision@N and nDCG@N results for the 2-class and 3-class relevance settings, together with corpus vocabulary statistics.

## Data Availability

All code, datasets and experimental results used in this study are publicly available through Zenodo to ensure full reproducibility. Each record includes a DOI and version control for long-term access, with setup instructions and documentation provided within each deposition. RELISH corpus and supporting datasets 10.5281/zenodo.14801641. whatizit-dictionary 10.5281/zenodo.15096055. word2doc2vec 10.5281/zenodo.15094699. doc2vec 10.5281/zenodo.15095071. wmd-word2vec 10.5281/zenodo.15095228. fastText 10.5281/zenodo.15095937. Hybrid approaches (pre-annotation, post-annotation, post-reduction) 10.5281/zenodo.15094064. BERT approaches 10.5281/zenodo.18969187. Docker setup 10.5281/zenodo.14865428. Evaluation results and output embeddings 10.5281/zenodo.14826813.

## Footnotes

1 https://github.com/piskvorky/gensim-data

2 https://github.com/facebookresearch/fastText

3 https://www.sbert.net

4 https://www.nlm.nih.gov/databases/download/mesh.html

## Declarations

### Use of artificial intelligence tools

ChatGPT (OpenAI) was used to assist with paraphrasing selected passages during manuscript preparation. All AI-generated output was reviewed, verified and revised by at least one co-author to ensure accuracy and consistency with the overall manuscript. The authors take full responsibility for the integrity of the final text.

## References

[1] Lipscomb CE. Medical subject headings (MeSH). Bull Med Libr Assoc. 2000;88(3):265–66. 10928714 PMC35238

[2] Brown P, Tan A-C, El-Esawi MA, Liehr T, Blanck O, Gladue DP, et al. Large expert-curated database for benchmarking document similarity detection in biomedical literature search. Database. 2019;2019:baz085. 10.1093/database/baz085. 10.1093/database/baz085 PMC7291946 33326193

[3] Pato M, Barros M, Couto FM. Survey on recommender systems for biomedical items in life and health sciences. ACM Comput Surv. 2024;56(6):1–32. 10.1145/3639047.

[4] TF–IDF. In: Sammut C, Webb GI, editors. Encyclopedia of machine learning. Boston, MA: Springer US; 2010. p. 986–87.

[5] Kumar NVA, Mehrotra S. A comparative analysis of word embedding techniques and text similarity measures. In: 2022 5th International Conference on Contemporary Computing and Informatics (IC3I). 2022. p. 1581–85. Available from: 10.1109/IC3I56241.2022.10072927.

[6] Bollacker KD, Lawrence S, Giles CL. CiteSeer: an autonomous web agent for automatic retrieval and identification of interesting publications. In: AGENTS ’98: Proceedings of the Second International Conference on Autonomous Agents. 1998. Available from: 10.1145/280765.280786.

[7] Achakulvisut T, Acuna D, Ruangrong T, Kording K. Science concierge: a fast content-based recommendation system for scientific publications. PLoS One. 2016;11(7):e0158423. 10.1371/journal.pone.0158423. 10.1371/journal.pone.0158423 PMC4934767 27383424

[8] Yoneya T, Mamitsuka H. PURE: a PubMed article recommendation system based on content-based filtering. In: Genome Informatics International Conference on Genome Informatics. 2007. p. 267–76. 10.1142/9781860949920_0026;18. 18546494

[9] Desai P, Telis N, Lehmann B, Bettinger K, Pritchard JK, Datta S. SciReader: a cloud-based recommender system for biomedical literature. bioRxiv. 2018. 10.1101/333922.

[10] Lin J, Wilbur W. PubMed related articles: a probabilistic topic-based model for content similarity. BMC Bioinf. 2007;8(1):423. 10.1186/1471-2105-8-423. 10.1186/1471-2105-8-423 PMC2212667 17971238

[11] Wang Y, Liu S, Afzal N, Rastegar-Mojarad M, Wang L, Shen F, et al. A comparison of word embeddings for the biomedical natural language processing. J Biomed Inf. 2018;87:12–20. 10.1016/j.jbi.2018.09.008. 10.1016/j.jbi.2018.09.008 PMC6585427 30217670

[12] Cichosz P. Active learning for biomedical article classification with bag of words and FastText embeddings. Appl Sci. 2024;14(17):7945. 10.3390/app14177945.

[13] Zhu Y, Yan E, Wang F. Semantic relatedness and similarity of biomedical terms: examining the effects of recency, size, and section of biomedical publications on the performance of word2vec. BMC Med Inf Decis Mak. 2017;17(1):1–8. 10.1186/s12911-017-0498-1. 10.1186/s12911-017-0498-1 PMC5496182 28673289

[14] Devlin J, Chang MW, Lee K, Toutanova K. BERT: pre-training of deep bidirectional transformers for language understanding. In: Proceedings of NAACL-HLT 2019. 2019. p. 4171–86. Available from: 10.18653/v1/N19-1423.

[15] Lee J, Yoon W, Kim S, Kim D, Kim S, So CH, et al. BioBERT: a pre-trained biomedical language representation model for biomedical text mining. Bioinformatics. 2020;36(4):1234–40. 10.1093/bioinformatics/btz682. 31501885 10.1093/bioinformatics/btz682 PMC7703786

[16] Beltagy I, Lo K, Cohan A. SciBERT: a pretrained language model for scientific text. In: Proceedings of EMNLP-IJCNLP 2019. 2019. p. 3615–20. Available from: 10.18653/v1/D19-1371.

[17] Zhang L, Lu W, Chen H, Huang Y, Cheng Q. A comparative evaluation of biomedical similar article recommendation. J Biomed Inf. 2022;131:104106. 10.1016/j.jbi.2022.104106. 10.1016/j.jbi.2022.104106 35661818

[18] Jiang S, Wu W, Tomita N, Ganoe C, Hassanpour S. Multi-Ontology Refined Embeddings (MORE): a hybrid multi-ontology and corpus-based semantic representation model for biomedical concepts. J Biomed Inf. 2020;111:103581. 10.1016/j.jbi.2020.103581. 10.1016/j.jbi.2020.103581 PMC7665985 33010425

[19] Chen Q, Lee K, Yan S, Kim S, Wei CH, Lu Z. BioConceptVec: creating and evaluating literature-based biomedical concept embeddings on a large scale. PLoS Comput Biol. 2020;16(4):e1007617. 10.1371/journal.pcbi.1007617. 10.1371/journal.pcbi.1007617 PMC7237030 32324731

[20] Zhang Y, Chen Q, Yang Z, Lin H, Lu Z. BioWordVec, improving biomedical word embeddings with subword information and MeSH. Sci Data. 2019;6(1):52. 10.1038/s41597-019-0055-0. 10.1038/s41597-019-0055-0 PMC6510737 31076572

[21] Brown P. RELISH_v1. Figshare. Dataset. Available from: 10.6084/m9.figshare.7722905.v1.

[22] Mikolov T, Chen K, Corrado G, Dean J. Efficient estimation of word representations in vector space. In: Proceedings of the International Conference on Learning Representations (ICLR). 2013. Available from: 10.48550/arXiv.1301.3781.

[23] Řehůřek R, Sojka P. Software framework for topic modelling with large corpora. In: Proceedings of the LREC 2010 Workshop on New Challenges for NLP Frameworks. 2010. p. 45–50. Available from: 10.13140/2.1.2393.1847.

[24] Kusner MJ, Sun Y, Kolkin NI, Weinberger KQ. From word embeddings to document distances. In: Proceedings of the 32nd International Conference on International Conference on Machine Learning - Volume 37. ICML’15. JMLR.Org. 2015. p. 957–66. Available from: https://proceedings.mlr.press/v37/kusnerb15.pdf.

[25] Le Q, Mikolov T. Distributed representations of sentences and documents. In: Proceedings of the 31st International Conference on Machine Learning (ICML). 2014. p. 1188–96. Available from: 10.48550/arXiv.1405.4053.

[26] Bojanowski P, Grave E, Joulin A, Mikolov T. Enriching word vectors with subword information. Trans Assoc Comput Linguist. 2017;5:135–46. 10.1162/tacl_a_00051.

[27] Cohan A, Feldman S, Beltagy I, Downey D, Weld DS. SPECTER: document-level representation learning using citation-informed transformers. In: Proceedings of ACL 2020. 2020. p. 2270–82. Available from: 10.18653/v1/2020.acl-main.207.

[28] Liu F, Shareghi E, Meng Z, Basaldella M, Collier N. Self-alignment pretraining for biomedical entity representations. In: Proceedings of the 2021 Conference of the North American Chapter of the Association for Computational Linguistics: Human Language Technologies. 2021. p. 4228–38. Available from: 10.18653/v1/2021.naacl-main.334.

[29] Rebholz-Schuhmann D, Arregui M, Gaudan S, Kirsch H, Jimeno A. Text processing through web services: calling whatizit. Bioinformatics. 2008, 11;24(2):296–98. 10.1093/bioinformatics/btm557. 18006544 10.1093/bioinformatics/btm557

[30] Gonçalves C, Gonçalves C, Camacho R, Oliveira E. The impact of pre-processing on the classification of MEDLINE documents. In: Proceedings of the 3rd International Conference on Bioinformatics. 2010. p. 53–61. Available from: https://www.scitepress.org/papers/2010/30287/30287.pdf.

[31] Ye X, Shen H, Ma X, Bunescu R, Liu C. From word embeddings to document similarities for improved information retrieval in software engineering. In: Proceedings of the 38th International Conference on Software Engineering (ICSE). 2016. Available from: 10.1145/2884781.2884862.

[32] Zhu S, Zeng J, Mamitsuka H. Enhancing MEDLINE document clustering by incorporating MeSH semantic similarity. Bioinformatics. 2009;25(15):1944–51. 10.1093/bioinformatics/btp338. 19497938 10.1093/bioinformatics/btp338

[33] Gero Z, Ho J. Pmcvec: distributed phrase representation for biomedical text processing. J Biomed Inf. 2019;100:100047. Articles initially published in Journal of Biomedical Informatics: X 1-4, 2019. 10.1016/j.yjbinx.2019.100047. 10.1016/j.yjbinx.2019.100047 34384576

[34] Akiba T, Sano S, Yanase T, Ohta T, Koyama M. Optuna: a next-generation hyperparameter optimization framework. In: Proceedings of the 25th ACM SIGKDD International Conference on Knowledge Discovery & Data Mining. KDD ’19. New York, NY, USA: Association for Computing Machinery; 2019. p. 2623–31. Available from: 10.1145/3292500.3330701.

[35] Reimers N, Gurevych I. Sentence-BERT: sentence embeddings using Siamese BERT-Networks. Proceedings of EMNLP-IJCNLP 2019. 2019. p. 3982–92. Available from: 10.18653/v1/D19-1410.

