# Supplementary material for: Benchmarking MeSH-augmented embeddings for biomedical document similarity
Source: J Biomed Semantics. 2026 Aug 14;17:16. doi: 10.1186/s13326-026-00365-6 (PMC13476922; doi:10.1186/s13326-026-00365-6)
Supplement: Supplementary file 1 [file 13326_2026_365_MOESM1_ESM.pdf]

Additional file 1 - Supplementary Tables

Approach	Training type	P@5	P@10	P@15	P@20
whatizit-dictionary	-	0.820	0.793	0.778	0.771
word2doc2vec	P	0.793	0.764	0.757	0.739
word2doc2vec	T	<u>0.815</u>	0.789	0.777	0.759
pre-annotation-word2doc2vec	T	0.797	0.775	0.759	0.748
post-annotation-word2doc2vec	T	0.812	0.788	0.774	0.764
postreduction-word2doc2vec	T	0.810	0.784	0.769	0.759
wmd-word2vec	P	0.777	0.759	0.755	0.746
wmd-word2vec	T	<u>0.813</u>	0.790	0.775	0.758
pre-annotation-wmd-word2vec	T	0.802	0.788	0.769	0.750
post-annotation-wmd-word2vec	T	0.802	0.776	0.766	0.753
postreduction-wmd-word2vec	T	0.792	0.775	0.762	0.750
fasttext	P	0.771	0.762	0.749	0.738
fasttext	T	0.800	0.778	0.767	0.757
pre-annotation-fasttext	T	0.793	0.767	0.765	0.750
post-annotation-fasttext	T	<u>0.808</u>	0.784	0.768	0.759
postreduction-fasttext	T	0.807	0.789	0.769	0.759
doc2vec	T	0.823	0.797	0.780	0.767
pre-annotation-doc2vec	T	<u>0.839</u>	0.812	0.791	0.777
BioBERT-base	P	0.782	0.765	0.746	0.740
BioBERT-base	F	**0.907**	**0.876**	**0.851**	**0.829**
BioBERT-large	P	0.802	0.769	0.760	0.754
BioBERT-large	F	0.890	0.860	0.841	0.822
SciBERT	P	0.793	0.766	0.759	0.748
SciBERT	F	0.875	0.859	0.838	0.813
SPECTER	P	0.864	0.839	0.815	0.800
SPECTER	F	0.879	0.865	0.843	0.832
SapBERT	P	**0.877**	**0.851**	**0.829**	**0.808**
SapBERT	F	0.887	0.865	0.848	0.835

Table S1: Precision@N under the 2-class relevance setting for all standard, hybrid and transformer-based approaches, evaluated under the 2-class relevance setting (*relevant + partially relevant* versus *non-relevant*). Training type: **P** indicates pre-trained models, **T** indicates models trained on the RELISH corpus and **F** indicates fine-tuned transformer models. For each column, the best overall score is shown in bold, and the best score within each approach category (groups separated by horizontal rules) is underlined.

Approach	Training type	P@5	P@10	P@15	P@20
whatizit-dictionary	-	0.512	0.479	0.455	0.449
word2doc2vec	P	0.485	0.448	0.430	0.409
word2doc2vec	T	<u>0.538</u>	0.481	0.460	0.444
pre-annotation-word2doc2vec	T	0.536	0.486	0.457	0.439
post-annotation-word2doc2vec	T	0.521	0.475	0.458	0.442
postreduction-word2doc2vec	T	0.530	0.483	0.457	0.441
wmd-word2vec	P	0.492	0.440	0.430	0.417
wmd-word2vec	T	0.526	0.475	0.455	0.436
pre-annotation-wmd-word2vec	T	0.528	0.484	0.450	0.431
post-annotation-wmd-word2vec	T	<u>0.533</u>	0.471	0.454	0.435
postreduction-wmd-word2vec	T	0.523	0.476	0.453	0.432
fasttext	P	0.461	0.433	0.415	0.407
fasttext	T	<u>0.536</u>	0.492	0.464	0.445
pre-annotation-fasttext	T	0.510	0.486	0.463	0.442
post-annotation-fasttext	T	0.530	0.489	0.462	0.441
postreduction-fasttext	T	0.533	0.490	0.456	0.439
doc2vec	T	0.515	0.481	0.463	0.443
pre-annotation-doc2vec	T	<u>0.516</u>	0.484	0.464	0.440
BioBERT-base	P	0.503	0.452	0.425	0.418
BioBERT-base	F	0.780	0.771	0.777	0.787
BioBERT-large	P	0.487	0.440	0.422	0.419
BioBERT-large	F	**0.799**	**0.790**	**0.794**	**0.807**
SciBERT	P	0.489	0.449	0.438	0.422
SciBERT	F	0.760	0.750	0.756	0.764
SPECTER	P	**0.603**	**0.551**	**0.511**	**0.484**
SPECTER	F	0.639	0.562	0.528	0.497
SapBERT	P	0.613	0.537	0.502	0.477
SapBERT	F	0.636	0.578	0.538	0.509

Table S2: Precision@N under the 3-class relevance setting for all standard, hybrid, and transformer-based approaches, evaluated under the 3-class relevance setting (*relevant* versus *partially relevant* versus *non-relevant*). Training type: **P** indicates pre-trained models, **T** indicates models trained on the RELISH corpus, and **F** indicates fine-tuned transformer models. For each column, the best overall score is shown in bold, and the best score within each approach category (groups separated by horizontal rules) is underlined.

Approach	Training type	nDCG@5	nDCG@10	nDCG@15	nDCG@20
whatizit-dictionary	-	0.642	0.646	0.658	0.681
word2doc2vec	P	0.617	0.620	0.634	0.646
word2doc2vec	T	<u>0.662</u>	0.658	0.667	0.684
pre-annotation-word2doc2vec	T	0.648	0.645	0.653	0.668
post-annotation-word2doc2vec	T	0.657	0.654	0.662	0.682
postreduction-word2doc2vec	T	0.655	0.652	0.657	0.676
wmd-word2vec	P	0.636	0.627	0.639	0.657
wmd-word2vec	T	<u>0.666</u>	0.666	0.672	0.684
pre-annotation-wmd-word2vec	T	0.665	0.665	0.666	0.679
post-annotation-wmd-word2vec	T	0.666	0.658	0.667	0.679
postreduction-wmd-word2vec	T	0.657	0.655	0.664	0.675
fasttext	P	0.611	0.617	0.626	0.647
fasttext	T	0.656	0.651	0.660	0.678
pre-annotation-fasttext	T	0.653	0.653	0.663	0.674
post-annotation-fasttext	T	0.656	0.651	0.660	0.678
postreduction-fasttext	T	<u>0.664</u>	0.663	0.669	0.683
doc2vec	T	0.648	0.655	0.667	0.682
pre-annotation-doc2vec	T	<u>0.660</u>	0.663	0.678	0.694
BioBERT-base	P	0.623	0.624	0.629	0.651
BioBERT-base	F	**0.777**	**0.760**	**0.769**	**0.781**
BioBERT-large	P	0.628	0.625	0.637	0.661
BioBERT-large	F	0.754	0.751	0.760	0.773
SciBERT	P	0.624	0.626	0.641	0.659
SciBERT	F	0.730	0.733	0.741	0.753
SPECTER	P	0.732	0.731	0.732	0.745
SPECTER	F	0.766	0.755	0.762	0.777
SapBERT	P	**0.746**	**0.735**	**0.741**	**0.753**
SapBERT	F	0.775	0.771	0.775	0.790

Table S3: nDCG@N for a 2-class distribution for all standard, hybrid and transformer-based approaches, evaluated under the 2-class relevance setting (*relevant + partially relevant* versus *non-relevant*). Training type: **P** indicates pre-trained models, **T** indicates models trained on the RELISH corpus and **F** indicates fine-tuned transformer models. For each column, the best overall score is shown in bold, and the best score within each approach category (groups separated by horizontal rules) is underlined.

Approach	class	algorithm	vector_size	window	min_count	epochs	model vocabulary size	OOV word count
word2doc2vec	2	sg	300	11	3	6	150,048	22,514
word2doc2vec	3	sg	350	12	2	15	209,317	20,419
wmd-word2vec	2	sg	100	12	1	10	429,032	17,170
wmd-word2vec	3	cbow	350	13	2	15	209,317	20,419
doc2vec	2	dm	250	6	1	13	429,032	-
doc2vec	3	dm	450	10	2	14	209,317	-
fasttext	2	sg	250	5	1	9	429,032	0
fasttext	3	sg	450	14	1	14	429,032	0
pre-annotation-word2doc2vec	2	sg	100	11	2	7	216,984	21,666
pre-annotation-word2doc2vec	3	sg	450	15	3	15	154,874	23,899
post-annotation-word2doc2vec	2	sg	250	12	1	11	457,200	17,170
post-annotation-word2doc2vec	3	sg	250	15	2	14	235,282	20,419
postreduction-word2doc2vec	2	sg	200	12	1	7	457,200	17,607
postreduction-word2doc2vec	3	sg	500	12	3	13	174,653	22,868
pre-annotation-wmd-word2vec	2	sg	150	7	2	11	216,984	21,666
pre-annotation-wmd-word2vec	3	sg	300	8	3	14	154,874	23,899
post-annotation-wmd-word2vec	2	sg	350	12	1	7	457,200	18,011
post-annotation-wmd-word2vec	3	cbow	100	6	1	15	457,200	18,011
postreduction-wmd-word2vec	2	sg	500	13	2	14	235,282	20,797
postreduction-wmd-word2vec	3	cbow	400	15	1	14	457,200	17,607
pre-annotation-doc2vec	2	dm	300	14	1	13	216,984	-
pre-annotation-doc2vec	3	dm	250	9	2	13	458,571	-
pre-annotation-fasttext	2	sg	350	7	3	15	154,874	0
pre-annotation-fasttext	3	sg	350	10	1	15	429,032	0
post-annotation-fasttext	2	sg	400	6	1	10	429,032	1910
post-annotation-fasttext	3	sg	500	14	1	12	429,032	1910
postreduction-annotation-fasttext	2	sg	400	7	1	13	429,032	1905
postreduction-annotation-fasttext	3	sg	450	15	3	6	150,048	1905

Table S4: Optimal hyperparameter configurations and vocabulary statistics for each standard and hybrid approach under the 2- and 3-class relevance settings. For each model the table reports the training algorithm (sg: skip-gram, cbow: continuous bag-of-words, dm: distributed memory), vector size, context window, `min_count` threshold, number of training epochs, the resulting model vocabulary size and the out-of-vocabulary (OOV) word count. Doc2Vec rows have no OOV count because document-level models do not rely on a fixed word vocabulary, and fastText shows near-zero OOV owing to its subword (n-gram) representation.
